# Wrinkling in engineering fabrics: a comparison between two different comprehensive modelling approaches

**DOI:** 10.1098/rspa.2018.0063

**Published:** 2018-08-08

**Authors:** I. Giorgio, P. Harrison, F. dell'Isola, J. Alsayednoor, E. Turco

**Affiliations:** 1 DISG, Università di Roma La Sapienza, Rome, Italy; 2 School of Engineering, University of Glasgow, Glasgow, UK; 3 Department of Architecture, Design and Urban planning, University of Sassari, Alghero, Italy; 4 International Research Center, M&MoCS, L'Aquila, Italy

**Keywords:** fabrics/textiles, elastic surface theory, second gradient models, wrinkling

## Abstract

We consider two ‘comprehensive’ modelling approaches for engineering fabrics. We distinguish the two approaches using the terms ‘semi-discrete’ and ‘continuum’, reflecting their natures. We demonstrate a fitting procedure, used to identify the constitutive parameters of the continuum model from predictions of the semi-discrete model, the parameters of which are in turn fitted to experimental data. We, then, check the effectiveness of the continuum model by verifying the correspondence between semi-discrete and continuum model predictions using test cases not previously used in the identification process. Predictions of both modelling approaches are compared against full-field experimental kinematic data, obtained using stereoscopic digital image correlation techniques, and also with measured force data. Being a reduced order model and being implemented in an implicit rather than an explicit finite-element code, the continuum model requires significantly less computational power than the semi-discrete model and could therefore be used to more efficiently explore the mechanical response of engineering fabrics.

## 1. Introduction

In the literature on the mechanical behaviour of engineering fabrics, several models and modelling approaches have been introduced. Among them, we focus our attention on two different ‘comprehensive’ modelling approaches; models able to independently control the tensile, shear, out-of-plane bending, in-plane bending and torsional stiffnesses of the fabric, with the stiffnesses convecting with the fibre directions during large shear deformations. So far, through-thickness rigidity is ignored but could also be introduced in future work (e.g. [1]). We refer to the two approaches as ‘semi-discrete’ and ‘continuum’ models, reflecting their semi-discrete and continuum-based natures.

The semi-discrete model [2,3] is based on the assumption that woven engineering fabrics can be modelled as a repeat unit cell, based on a pantographic module consisting of mutually constrained beam and membrane elements connected via zero-torque hinges. Fibre directions are tracked within the membrane elements [4], though the implemented constitutive model [5] contributes towards only the shear stiffness of the sheet while tensile, out-of-plane bending, in-plane bending and torsional stiffness are all modelled using beam elements; homogenization theory facilitates decoupled control over each individual stiffness parameter. This is clearly not the most detailed model which one could introduce. It is possible to conceive of ‘micro’ models; treating each fibre in the fabric as a three-dimensional continuum having a cylindrical reference configuration and interacting via contact with surrounding fibres (e.g. [6,7]). Still, in [2] it is shown that the computational burden required by what we call the semi-discrete model is already rather heavy, therefore ‘micro’ models are currently not viable for most practical applications, unless incorporated in a specifically adapted homogenization procedure. In many cases, ‘micro’ models will produce exactly the same results as the semi-discrete model and we therefore limit this evaluation to the predictions of the latter.

The continuum model is a second gradient continuum model. Such continua are characterized by a deformation energy which depends on both the first- and second-order displacement gradients and cannot be framed in the postulation scheme usually used in continuum mechanics (e.g. [8–12]). Here the standard concept of stress, strain and contact action are generalized [13–17]. In particular, we consider a two-dimensional continuum which generalizes standard plate theories using so called ‘geodesic’ curvatures (e.g. [18–21]), i.e. the curvature of the coordinate lines representing the actual configuration of the fibre directions in the fabric appear as an independent explicit variable in the function giving the deformation energy per unit area. Of course, we will not use the most general expression for such energy density but instead a specialized form, accounting for material symmetries. There are great difficulties in the identification of macro constitutive parameters in terms of meso- or micro-scale equivalents [22–26] and a general procedure leading to rigorous mathematical results is yet to be found. Therefore, we limit ourselves to identify the constitutive parameters of the continuum model by means of a ‘global’ fitting procedure, which considers the deformation of the whole body: we refrain from trying to identify the macro coefficients in terms of the geometrical and mechanical properties of the periodic semi-discrete cell. This global procedure uses a sufficiently large set of target predictions generated using the semi-discrete model to calibrate the mechanical parameters of the continuous macro-scale simulations. The parameters of the continuum model are chosen to provide the best fit with: (i) the shape of the deformed specimen under given displacements and (ii) the reaction force versus displacement produced by the specimen. Subsequently, we demonstrate that after this identification process, the continuum model is capable of producing accurate predictions well beyond the limited set of cases used in the fitting process. Indeed, verification of both models is provided by comparison with experimental data (measured on a twill-weave carbon fabric) [3]. The move from semi-discrete to macro-scale continuum modelling is useful due to potential advantages in terms of faster simulations (or reduced computational resource), simpler meshing and the potential for employing numerical techniques such as adaptive meshing. Encouraging results produced during this investigation are thought to justify the use of the continuum model despite recourse to a more complex conceptual framework (i.e. a more complex class of continuum models) and the requirement for a suitably adapted numerical integration scheme (based on higher-order shape functions).

Both modelling approaches are comprehensive and are therefore capable of accurately describing out-of-plane deformations. Such wrinkles can be regarded as low-energy states in comparison with purely planar ones. Using second gradient pantographic sheets, we can describe buckling phenomena in which out-of-plane wrinkling is influenced by rigidities related to geodesic curvature; these phenomena cannot be accounted for by standard plate models.

The contents of the paper are organized as follows. After a short introduction, in §2 the experimental arrangement used to validate the numerical predictions of the proposed models is described. In §3, an overview of both modelling approaches considered is provided. In §4, a semi-discrete/continuum numerical identification procedure is performed in order to evaluate the material parameters of the continuum model. Finally, a discussion of the results and conclusions are supplied in the last section.

## 2. Experimental method

### (a) Material

**Figure 1.**
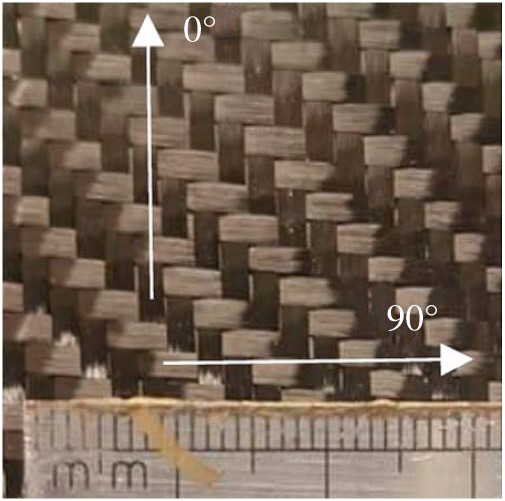
Twill-weave carbon fabric showing warp (0°) and weft (90°) tows and scale rule. (Online version in colour.)

The forming kinematics and mechanics of a 2 × 2 twill-weave carbon fabric (EasyComposites, product code = CF-22-20 0-150), treated with a speckle pattern for analysis using Digital Image Correlation (DIC) was characterized and reported in [3]. The width of warp and weft tows in the carbon fabric is 2.00 ± 0.01 and 1.92 ± 0.05 mm, respectively (figure 1). At a low compressive stress of 1 kPa, the thickness of the carbon fabric is 0.35 ± 0.01 mm when measured using a single layer and 0.30 ± 0.02 mm when measuring four stacked layers. At 100 kPa, the fabric was compressed to around 0.24 ± 0.01 mm per layer when measuring four stacked layers. The areal density was 203.1 ± 1.25 g m^−2^ and 210.9 ± 5.5 g m^−2^ before and after treatment for analysis using stereoscopic DIC (see §2c).

### (b) Mechanical forming properties

The mechanical forming properties were determined using a six-step procedure, discussed in detail in [3] and summarized here. The data provided refer to the semi-discrete model which we consider as a refined model (tables 1 and 2).

(i) For materials with an ill-defined thickness, such as engineering fabrics, the *tensile line stiffness* can be more easily measured than Young's modulus [27]. In this investigation, the tensile line stiffness per unit width in each fibre direction was chosen to be high enough to ensure that tensile strains were limited to less than 1% in the Uniaxial Bias Extension (UBE) simulations but low enough to allow reasonable simulation times using the explicit finite-element method (600 kNm^−1^ is usually a good compromise and is used in this investigation). Any dependence of the tensile modulus on tensile strain due to the effects of straightening of tow crimp, e.g. [28], was neglected.
(ii) The *out-of-plane bending stiffness* per unit width in each fibre direction was determined directly from experimental cantilever bending tests (with the test specimens cut along the warp and weft directions). Possible, nonlinear dependence of the bending stiffness on bending curvature was ignored [29]. A value of 0.0003128 Nm in the warp and 0.0002438 Nm in the weft direction were found.
(iii) The *shear force per unit length* was determined directly via a modified version of the usual UBE test using stress-power normalization theory [30]. The modification involves bonding aluminium to the test specimen in order to prevent intraply-slip and to create an ‘encastre’ boundary condition at the edge of the fabric [2,3]. Three different specimen sizes (100 × 200, 150 × 300 and 200 × 400 mm) were used and two tests were conducted on each specimen size. The average unintended pre-shear angle of the six test specimens in this investigation, when installed in the test machine, just prior to testing was −0.48° and the standard deviation of the pre-shear angle was 2.2° (these low values are a good pre-requisite for reliable data [31]). Possible coupling between the shear stiffness and the tensile stress acting along the tow directions was ignored (e.g [32]). The coefficients of the ninth-order polynomial fitted to the shear force versus shear angle curve ($F_{\mathrm{sh}}(\theta )=\sum_{i=1}^{9}a_{i}\;\theta^{i}$) are provided in table 2.
(iv) The tensile stiffness, out-of-plane bending stiffness in the tow directions and the shear stiffness were set in steps 1–3 and the in-plane bending stiffness was initially estimated as 2 × the out-of-plane bending stiffness. The *torsional stiffness of the unsheared* specimen was then determined by simulating the cantilever bending test with the test specimens cut along the bias direction. A mutually constrained pantographic beam and membrane mesh together with the stress-power model [5] was used to simulate the test (see §3b). The torsional stiffness per unit width in each fibre direction was adjusted until the observed and predicted bend angle matched to within a given tolerance, in this case 1.5%. Possible, nonlinear dependence of the torsional stiffness on the degree of twist was ignored.
(v) The *in-plane bending stiffness* per unit width in each fibre direction was next found by matching the simulated and measured shear kinematics of a UBE test. Specifically, by adjusting the in-plane bending stiffness, the shear angle predicted at the centre of the UBE specimen could be matched with experimental observations. This behaviour is specimen size dependent and so matching the kinematics with measurements on three different specimen sizes improved confidence in the measurements. Value of 0.0006 Nm and 0.00048 Nm in the warp and weft directions were determined.
(vi) Finally, the *torsional stiffness of the sheared specimen* was determined. A notable observation when conducting a UBE test is that the specimen often (but not always) wrinkles towards the later stages of the test. This wrinkle leads to a twisting of the tows in the fabric and can therefore be used to infer the torsional stiffness during shear. This value was determined via inverse modelling of the UBE test; the torsional stiffness was adjusted until the predicted and observed wrinkle onset angle matched. A value of 0.00002424 Nm and 0.000020353 Nm were found for the warp and weft directions.

**Table 1.** Stiffnesses evaluated for the semi-discrete model.

	tensile line (kNm^−1^)	out-of-plane bending (Nm)	in-plane bending (Nm)	torsional (Nm)
warp	600	3.128 × 10^−4^	6.0 × 10^−4^	2.424 × 10^−5^
weft	600	2.438 × 10^−4^	4.8 × 10^−4^	2.035 × 10^−5^

**Table 2.** Coefficients of ninth-order shear force versus shear angle polynomial input curve.

*a*_1_	0.5926 N	*a*_4_	−0.001593 N	*a*_7_	3.495 × 10^−8^ N
*a*_2_	−0.1416 N	*a*_5_	7.463 × 10^−5^ N	*a*_8_	−3.167 × 10^−10^ N
*a*_3_	0.01998 N	*a*_6_	−2.100 × 10^−6^ N	*a*_9_	1.204 × 10^−12^ N

Note that torsional stiffness of a sheet is its resistance to twisting. This property has long been recognized in modelling apparel [33] but rarely considered in the field of engineering textiles. It is clear that if a tow has a torsional stiffness [34] then an array of tows, organized in a sheet, must also have a torsional stiffness, though the relationship between the meso and macro torsional stiffness is complicated by the nature of the meso-structure. Indeed, roughly speaking, the torsional stiffness of the whole fabric is due not only to the torsional resistance of constituting fibres but also to their bending resistance.

### (c) Digital image correlation

UBE test specimens were treated to enable DIC analysis of the deformation. The treatment involved brushing the surface with graphite powder to reduce the reflectivity of the carbon fabric, before applying a speckle pattern by sputtering the graphite-treated surface with high viscosity white paint (e.g. Pebeo Acrylic); some degree of fabric stiffening is introduced at this stage [3]. A VIC 3-D DIC system was used with a two video camera set-up. VIC-3D 2010 software (Correlated Solutions, 2010) was used to analyse the videos. A kernel size of 14 × 14 and Gaussian smoothing were used to analyse the data. The VIC-3D 2010 software provided the *x*–*y*–*z* nodal coordinates of points on the surface of the fabric at different time increments during each test. Three-node triangular elements together with linear shape functions were used to represent the three-dimensional surface. The commercial system can provide several strain measures from the raw data but does not directly provide the fabric shear angle; a commonly used measure of strain when characterizing the forming mechanics of engineering fabrics. An in-house code was written to extract these data from the cloud of positional data points provided by the commercial software. The technique used to track the two-dimensional tow directions is similar to that outlined in [35], itself based on earlier work by Peng & Cao [36]. In this investigation, quaternion rotations are used to extend the two-dimensional algorithm to provide the shear angle following three-dimensional (out-of-plane) deformations. For details of the algorithm, see Alsayednoor *et al.* 2017 [31]. The low average initial pre-shear angle of the tests meant that non-orthogonal initialization of the tow directions when performing the DIC analysis [31] provided very little improvement of the data and so, for simplicity, was not applied to the data in this investigation.

## 3. Modelling approaches

### (a) The continuum model: a second gradient deformation energy model

The continuum model is a two-dimensional elastic surface that accounts for deformation in a three-dimensional Euclidean space. The simulations performed using the macro deformations model (and the consequent semi-discrete/continuum identification procedure and also the related comparisons with experimental evidences) are simply based on the method of minimization of the total energy of the system to characterize stable equilibrium configurations. Equilibrium (stable and unstable) configurations are searched among stationary configurations for the variation of total energy: however no appeal is made to balance equations, strong stationarity conditions or Euler–Lagrange conditions for stationarity. The numerical methods simply look for the stationarity of the calculated total energy, expressed in terms of a finite number of Lagrangian parameters approximately describing the configuration of the system. Therefore, the complete description of the continuous model is simply obtained by specifying the set of admissible displacement functions characterizing the shapes of the specimens and by postulating a suitable expression for deformation energy. We will not consider any equilibrium problem in which externally applied actions are imposed. Instead we will consider only equilibrium problems in which some displacements and normal gradients of displacements are assigned, which are enough to impose that rigid motions and floppy modes (i.e. all the displacement fields whose deformation energy is vanishing [37,38]) are also vanishing.

Such energy includes deformation energy, which we claim must depend also on second gradient of displacements [8,11,20,39], and the energy due to external actions. The structure of internal actions which can be sustained by second gradient materials is richer than the one corresponding to first gradient materials and, therefore, our model allows for the description of a wider range of deformation phenomena. We claim that this is needed in order to account for the presented experimental evidence. The reader should not be surprised by this circumstance. Such circumstance is often regarded as a problem if one does not accept to enlarge the scheme of continuum mechanics beyond first gradient continua. Many authors, indeed, seemed to believe that external actions applicable to a continuum should not depend on its nature, i.e. the properties of its deformation energy. It is not clear why this criticism should not be applied also to Euler fluids, which, as it is universally accepted, cannot sustain shear boundary forces.

In this paper (as done also in [40–42]), we consider a pantographic sheet. Its reference configuration can be identified as a suitably regular subset *B* of a plane and its generic shape as a suitably regular function **r** defined in *B* and having values in the Euclidean three-dimensional space. In the reference configuration, the pantographic sheet has two preferred directions, which are the directions of the fibres constituting it. We will assume nearly exclusively in this paper that they are orthogonal in the reference configuration (even if interesting theoretical and experimental results are available when this assumption is not verified, e.g. [43]) and we denote them with the symbols {**L, M**}.

The fibre stretches {λ, *μ*} and fibre trajectories {**l, m**} induced by the deformation are defined as follows:

λl=(∇r)Lμm=(∇r)M.

Because {**L, M**} is an orthonormal basis we may conclude that (see [11,20] for more details about all the following formulae)

∇r=λl⊗L+μm⊗M.

Moreover, the shear deformation angle and the deformed area density are given by

sin⁡γ:=l⋅m

and

J:=λμ|cos⁡γ|.

Consequently, the Cauchy–Green deformation tensor according to the fibre decomposition can be expressed as

$$C=\lambda^{2}L⊗L+\mu^{2}M⊗M+\lambda \mu \sin\gamma (L⊗M+M⊗L).$$

In order to introduce an objective expression for second gradient deformation energy related to the deformations of the fibres constituting pantographic sheets we need to introduce some useful vector and geometrically relevant scalar fields. With

p:=n×landq:=n×m,

we denote the vectors which are tangent to the curves orthogonal to the fibres on the deformed surface, being **n** the unit vector normal to the surface. By means of simple calculations, based on the differential geometry of embedded surfaces, we get the following representation for the second gradient of the placement field:

$$\nabla \nabla r=(g_{l}+K_{L}n)⊗L⊗L+(g_{m}+K_{M}n)⊗M⊗M+(\Gamma +Tn)⊗(L⊗M+M⊗L),$$

where

$$K_{L}:=\lambda^{2}\kappa_{l},\;K_{M}:=\mu^{2}\kappa_{m},\;T:=\lambda \mu \tau ,$$

being *κ*_*l*_ and *κ*_*m*_ the normal curvatures of the deformed fibres and *τ* the twist of the deformed surface, and where, being denoted *η*_*l*_, *η*_*m*_ the geodesic curvatures of the deformed fibres, and *ϕ*_*l*_, *ϕ*_*m*_ the so-called Tchebychev curvatures (e.g. [20]), we used the following definitions:

$$\begin{array}{rl} g_{l} & :=\lambda^{2}\eta_{l}p+(L\cdot \nabla \lambda )l,\;g_{m}:=\mu^{2}\eta_{m}q+(M\cdot \nabla \mu )m.\\ \Gamma & :=(L\cdot \nabla \mu )m+\lambda \mu \phi_{m}q=(M\cdot \nabla \lambda )l+\lambda \mu \phi_{l}p. \end{array}$$

By using the aforementioned definitions, it is possible to postulate an expression for deformation energy which respects the objectivity requirements and which, although being quadratic in the deformation measures, includes all geometric nonlinearities. Moreover, we will limit our attention to deformation energies which account for the material symmetry properties implied by the presence of two families of fibres. Moreover, we will assume that the fibres of considered pantographic sheets are constrained in such a way that they must be displaced by the same displacement field and that their relative rotation can only be a simple rotation around the normal of the actual shape. We are aware that these assumptions limit considerably the applicability range of the introduced continuum macro model, but it will be shown that it can capture some interesting and relevant phenomenological evidence.

The total deformation energy will be assumed to be composed by three terms, as follows:

3.1 $$W_{T}=W_{\mathrm{II}}+W_{e}+W_{s},$$

in which we introduced, respectively, the second gradient deformation energy associated with the bending, twisting and gradient of elongations of the constituting fibres (denoted by *W*_II_), the elongation deformation energy *W*_e_ and the shear deformation energy *W*_s_. More precisely, we assume that the second gradient deformation energy is given by

3.2 $$W_{\mathrm{II}}=\frac{1}{2}(A_{L}|g_{l}{|}^{2}+A_{M}|g_{m}{|}^{2}+A_{\Gamma }|\Gamma {|}^{2}+k_{L}K_{L}^{2}+k_{M}K_{M}^{2}+k_{T}T^{2}),$$

where *A*_*L*_, *A*_*M*_, *A*_*Γ*_, *k*_*L*_, *k*_*M*_ and *k*_T_ are suitable positive elastic coefficients which give the level of stiffness related to the deformation measure on which they act. Also we assume that

3.3 $$W_{e}=\frac{1}{2}(E_{L}\varepsilon_{L}^{2}+E_{M}\varepsilon_{M}^{2})$$

and

3.4 $$W_{s}(\gamma ,J)=G_{1}\left[{\left(1+{\left(\frac{\gamma }{\gamma_{0}}\right)}^{2}\right)}^{\beta }-1\right]-G_{2}(\log(J-J_{0})-\log(1-J_{0})-J+1),$$

where *J*_0_ is an incompressibility ‘threshold’ representing an area which cannot be attained with a finite deformation energy,

$$\varepsilon_{L}=\frac{1}{2}(\lambda^{2}-1)\;\mathrm{and}\;\varepsilon_{M}=\frac{1}{2}(\mu^{2}-1),$$

are the extensional fibre strains, *E*_*L*,*M*_ and *G*_1,2_ are positive elastic constants and *β* is a suitable positive power. Note that the expression for *W*_s_ is suggested by adapting in the present context the general considerations presented in [44]. Here, we assume there are no floppy modes simply because of the presence of the shear contribution to the energy given by *W*_s_. If we remove such a term, there is no energy associated to the macro shear of the fabric (the two family of fibres can rotate freely without storing any elastic energy), and then we have a floppy mode: i.e. a macro deformation with no energy.

At the macro-level, the kinematic boundary conditions which can be imposed for second gradient materials may also involve normal first derivatives of displacement at the boundaries and natural boundary conditions that involve correspondingly not only external forces but also double forces (e.g. [8,20]). These last boundary conditions can be evaluated starting from their microscopic interpretation. Indeed, double forces could originate by torques applied to the fibre ends at a microscopic scale, which in the continuum limit become a distributed action on the edge. On the other hand, if two opposite forces are applied at two adjacent points of the fabric microstructure, they result in a macro deformation, a dilation, which can be taken into account by the presence of double forces at macro level (see for more details [45,46]).

It would be desirable to deduce rigorously an expression for deformation energy of the form postulated here by starting from a semi-discrete model involving structures constituted by three-dimensional beams interconnected by suitable constraints and by membranes: this may require a remarkable mathematical effort. Some results in attacking this problem are already available in the literature [12,39]. In the present paper, the semi-discrete/continuum identification procedure is obtained qualitatively in this section via heuristic considerations leading to a direct postulation of the continuum model, reflecting the mechanical and geometrical properties of the semi-discrete model. In the following section (§4) a quantitative identification is based on targeted numerical simulations and a best-fit choice of the macro elastic constitutive parameters introduced in the present section is obtained.

### (b) The semi-discrete model: a beam lattice approach

**Figure 2.**
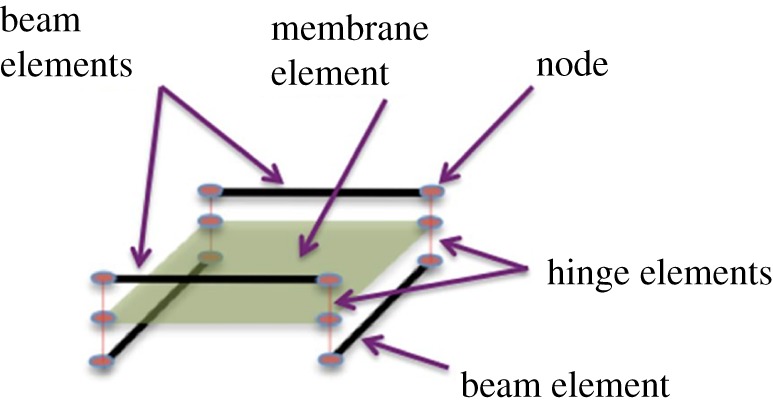
Repeat unit cell structure of the mutually constrained pantographic beam and membrane mesh, image reproduced from [2]. (Online version in colour.)

The semi-discrete model introduced in [2,3] is intended to supply an ‘overall’ description of the mechanical behaviour of a ‘physical’ mesh of the considered fabric. The basic twill-weave fibres structure is modelled as two pairs of parallel beams being interconnected at their extremity (figure 2), by internal hinges (nodes). The changes of area of this elementary structure must need the storage of deformation energy, as it is obvious when one thinks to the physical structure of the modelled fabric (figure 1). Therefore, a membrane element is introduced connected to the previously introduced quadrilateral elementary beam lattice.

This model can be called ‘semi-discrete’ as, at least in equilibrium, the deformation energy of each cell is known when the displacements of the four nodes (figure 2) and the rotations of the beams in these nodes are known. This means that the knowledge of a discrete set of Lagrangian parameters is sufficient to calculate the whole deformation energy of the considered system: of course the presence of the beam and membrane elements justifies the use of adjective ‘semi’ used in the section title.

The semi-discrete finite-element simulations involved use of the ‘mutually constrained pantographic beam and membrane mesh’ described in [2], implemented in Abaqus Explicit^TM^. The mesh comprises beam elements of length, *l*, and of rectangular cross section with thickness, *t*_*i*_, and width, *w*_*i*_, where the subscript *i* equals 1 (warp) or 2 (weft), and square membrane elements, also of side length, *l*, connected via hinge elements (figure 2). Specifically, we used B31 beam element and M3D4R membrane elements connected by hinge elements.

This mesh structure means that the elements translate together (mutually constrained) while permitting torque-free rotation of the two, initially perpendicular sets of beam elements, similar to a pantograph [47,48]. Homogenization theory relating the macro-scale mechanical properties (tensile, flexural and torsional stiffness) and areal density of the fabric, to the properties (physical density, stiffness and cross section) of the structural elements within the mesh was presented in [2,3]. The goal of the homogenization theory is to allow the macro-scale mechanical properties of the sheet to be independently set, and to dissociate these properties from the element length within the mesh (i.e. the mesh density). To achieve this, the beam stiffness, *E*_*i*_ and the beam cross section (thickness, *t*_*i*_ and width, *w*_*i*_) were related to the tensile line-stiffness per unit width, *γ* [27], the out-of-plane bending stiffness per unit width, *β* the in-plane bending stiffness per unit width, *α*, the specimen/blank width, *W*, and the beam element length, *l*, as

3.5 $$E_{i}=\frac{\gamma_{i}^{2}Wl}{12(W+l)\sqrt{\alpha_{i}\beta_{i}}},\;t_{i}=\sqrt{\frac{12\beta_{i}}{\gamma_{i}}}\;\mathrm{and}\;w_{i}=\sqrt{\frac{12\alpha_{i}}{\gamma_{i}}},$$

where the subscript, *i*, indicates the orientation of the set of beam elements, in either the 1 (warp) or the 2 (weft) direction. The torsional stiffness per unit width in each fibre direction can also be written as

3.6 $$\mu_{i}=\{\begin{array}{cc} 3\frac{\Gamma_{i}}{\xi_{i}}\beta_{i} & \mathrm{if}\;\beta_{i}<\alpha_{i}\\ 3\frac{\Gamma_{i}}{\xi_{i}}\alpha_{i} & \mathrm{if}\;\alpha_{i}<\beta_{i}, \end{array}$$

where *Γ* is a dimensionless numerical constant given by Saint Venant's analysis of the torsion of rectangular beams and depends on the aspect ratio between the short and long side lengths of the cross section of the beam. Relationships between the density of the beam and membrane elements to the areal density of the fabric are also provided in [2]. *ξ*_*i*_ is a dimensionless parameter, used to uncouple the torsional stiffness from the flexural behaviour of the sheet.

## 4. Quantitative semi-discrete/continuum numerical identification procedure

Owing to the robust performance of the explicit finite-element method in solving problems with complex contact conditions, the semi-discrete/continuum used in this investigation has been implemented in Abaqus Explicit (with a goal of using the model in analysing complex forming simulations in future). Nevertheless, it is well known that combining high moduli and low densities in the explicit finite-element method [49] can lead to long run times unless either mass or time-scaling are employed. In [2], wrinkling behaviour was shown to be strongly influenced by inertial effects, severely restricting the use of mass and time-scaling when investigating the form of developing wrinkles. This, coupled with the use of translational and rotational constraints (hinge elements) in the semi-discrete model, inevitably leads to long runs times (1 or 2 days when using a single workstation or a few hours when using supercomputing facilities and multiple licence tokens). By contrast, corresponding simulations performed with the continuum model, which uses an implicit finite-element solver (COMSOL Multiphysics^TM^) and shell Argyris elements (P5-triangle), requires less than 1 h to reach completion on a single workstation. We use different finite-element softwares, in modelling the continuum and semi-discrete approaches simply because the paper was the fruit of a collaboration between two independent groups. In the light of what has been said above, it is useful to determine the constitutive parameters appearing in the second gradient deformation energy by matching the kinematic predictions and mechanical properties of the semi-discrete and continuum models. Once the fitting process is completed, and the results verified using further test cases (beyond those used in the fitting process), the continuum model then can be used to more efficiently explore a wide range of alternative deformations. Parameter identification was conducted as follows: given a set of numerical predictions obtained from the semi-discrete model we conduct a further series of simulations using the continuum model, looking for the specific set of continuum parameters which best reproduce a specific global behaviour obtained from the semi-discrete simulations. The range in which we search for the best-fitting continuum parameters is suggested by heuristic considerations, leading to the continuum model described in §3a. For instance, we postulate that the fibres can be regarded as beams and therefore use as a first tentative value for the out-of-plane bending stiffnesses, *k*_*L*_ and *k*_*M*_, the corresponding bending stiffnesses given by classical beam theory, weighted by the density per unit length of fibres which constitute the pantographic sheet. This value gives an approximate first-order estimate of the final result. Once the best fit is obtained for the force–displacement plot, we use the obtained values for determining the shape of the pantographic sheet using the continuum model and we have compared with the shapes calculated with the semi-discrete model, in particular in the regimes in which the pantographic sheet remains planar. Finally, we considered equilibrium shapes in which wrinkles are formed. What is promising, and suggests that the range of applicability of the continuum model is even wider than expected, is that, *a posteriori*, once the continuum parameters for a given semi-discrete model are determined on the basis of few numerical simulations, then the continuum model still supplies predictions very close to those produced by the semi-discrete model.

### (a) Tensile results

Following the procedure summarized in §2b, we consider a first numerical experiment to determine the tensile properties along fibre directions. A tensile test on a rectangular sample whose fibres are disposed along the directions of the edges and with the size 0.115 × 0.025 m is performed employing the continuum model to this aim. In particular, the simulation is performed pulling the sample in the direction of the larger edge until a displacement of 0.00115 m, to get a strain of 1%. Indeed, in this range of small deformation the behaviour can be assumed linear and then we need to evaluate only the slope in the stress versus tensile strain plot. To reproduce the same force versus displacement plot obtained from the semi-discrete simulations, we determine a value of 5.4 × 10^5^ Nm^−1^ for the elongation stiffness (*E*_*L*_ = *E*_*M*_) related to the continuum model.

### (b) Cantilever results with specimens cut along the warp and weft directions

To evaluate the out-of-plane bending stiffnesses, test specimens cut along the warp and weft directions have been used to perform cantilever bending tests in which a fabric strip is subject to deformation due to its own weight. We consider a rectangular sample of size *L* = 0.118 m and *w* = 0.02 m with aspect ratio λ = *L*/*w* = 5.9 whose mass density for unit area is assumed to be 0.2109 kg m^−2^.

**Figure 3.**
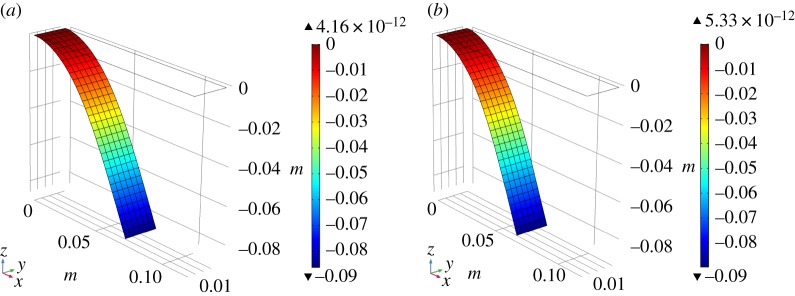
Deformed cantilever strip. Colour indicates the out-of-plane displacement. Long fibres along (*a*) warp and (*b*) weft directions. (Online version in colour.)

In these tests, the global behaviour which we use as target function to closely match the semi-discrete model predictions with the continuum model simulations is the angular deflection of the cantilever end, i.e. *ϕ* [2]. Specifically, we conduct two numerical simulations with long fibres arranged along the warp and weft direction (see figure 3*a* and *b*, respectively). In the first case, to obtain the same angular deflection, *ϕ* = 49.8°, of the semi-discrete simulation, we set an out-of-plane stiffness equal to *k*_*L*_ = 3 × 10^−4^ Nm. In the same way for the other case, we evaluate an out-of-plane stiffness equal to *k*_*M*_ = 2.6 × 10^−4^ Nm, to reproduce the angular deflection *ϕ* = 53.0° obtained from the semi-discrete model.

### (c) Uniaxial Bias Extension results

Subsequently, we perform UBE tests on three rectangular samples cut along the bias direction. The smallest sample is *L* = 0.2082 m long and *w* = 0.1019 m wide with aspect ratio λ = *L*/*w* = 2.043. To get the dimensions of the larger samples simply scale, these dimensions by 1.5 × and 2 ×.

#### (i) Axial force and shear angle predictions

**Figure 4.**
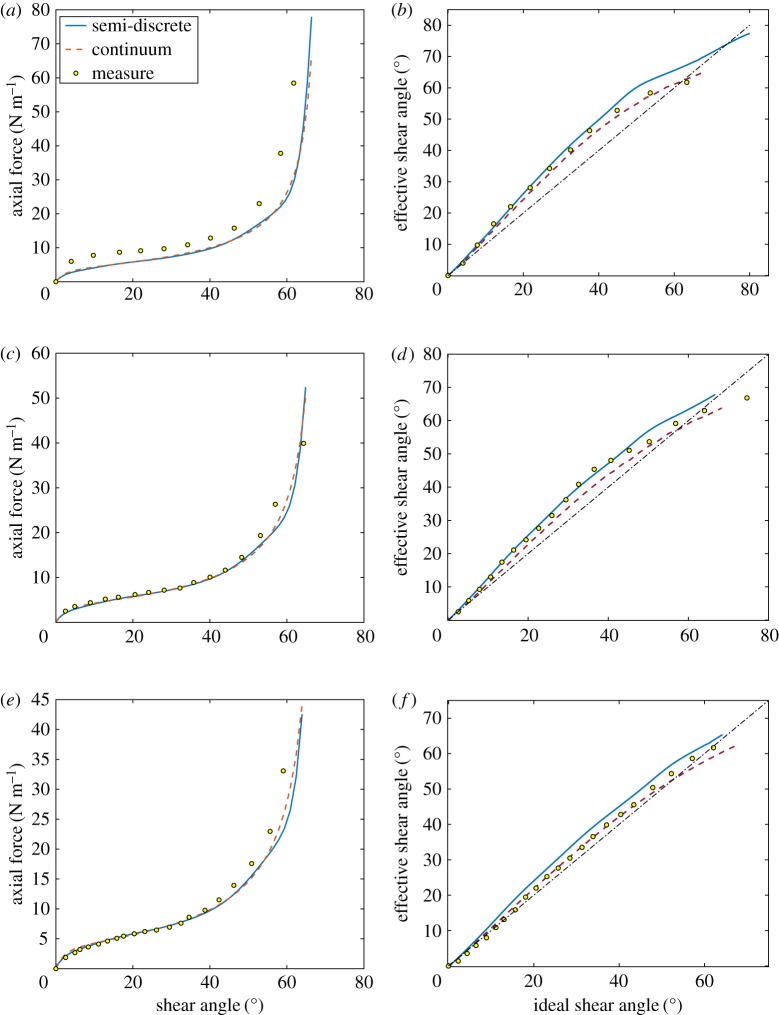
UBE test for the samples of size: (*a*,*b*) 100 × 200 mm; (*c*,*d*) 150 × 300 mm; (*e*,*f* ) 200 × 400 mm. (*a*,*c*,*e*) Normalized axial force versus shear angle. (*b*,*d*,*f* ) Effective shear angle versus ideal shear angle. (Online version in colour.)

To estimate the (trellis) shear resistance of the sheet, we fit the force versus displacement plots obtained from the semi-discrete simulations of UBE tests (itself fitted to experimental results, also shown in figure 4) with the corresponding plots predicted by the continuum model. Figure 4 includes both experimental tests results (originally presented in [3]) together with predictions obtained using the two distinct modelling approaches. The axial force is normalized by the side length of the central region [3] (0.0720 m for the smallest sample) and is plotted against the shear angle predicted at the centre of the sample. The shear parameters of the continuum model was adjusted until the data predicted by the semi-discrete model matched to within a given tolerance that of the semi-discrete model. Specifically, we obtain *G*_1_ = 0.0284 N m^−1^, *G*_2_ = 0.7406 N m^−1^, *γ*_0_ = 0.0304, *β* = 0.5806 and *J*_0_ = sin(*π*/9).

**Figure 5.**
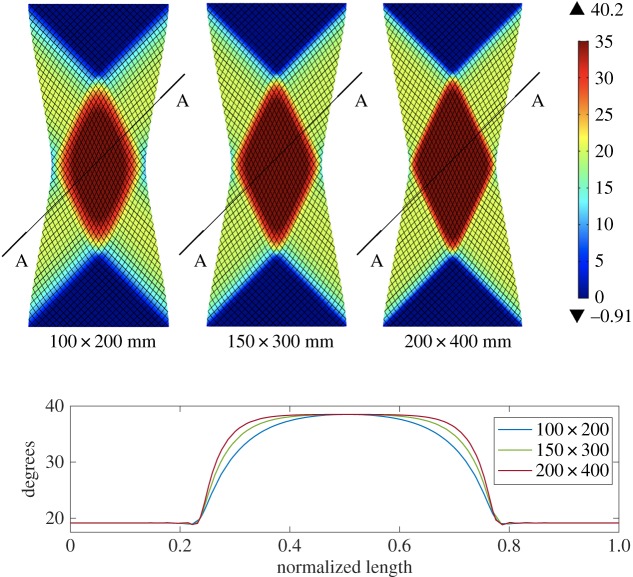
UBE test for the considered samples. Colours indicate shear angles. The plot shows the graphs of the shear angles along the cuts AA. (Online version in colour.)

Figure 5 shows the equilibrium shapes predicted for the three UBE specimen sizes at an imposed displacement which gives a shear angle at the centre of 40°. The figure can be compared with fig. 14 in [3]. The notable point about this image (and also fig. 14 in [3]) is the dependence of the full-field shear angle kinematics on the specimen size. This size-dependence is a direct consequence of using a second-order gradient approach to modelling the fabric (or according to the interpretation of Harrison *et al.* [3], is a result of introducing an in-plane bending stiffness into the fabric's constitutive response). Similar specimen size dependence was observed in the experiments conducted in [3], and is visible in the kinematic plots of figure 4*b*,*d* and *f* (discussed in the next section).

#### (ii) Shear angle at centre of specimen

The UBE tests allow us also to determine the in-plane bending stiffnesses using as target function for the best fit the actual shear angle at the centre of specimen versus the ideal shear angle, see figure 4*b*,*d* and *f* for the samples of size 100 × 200 mm, 150 × 300 mm and 200 × 400 mm, respectively. In particular, we determine *A*_*L*_ = 6 × 10^−4^ Nm, *A*_*M*_ = 5.2 × 10^−4^ Nm and *A*_*Γ*_ = 1.89 × 10^−4^ Nm.

### (d) Cantilever results with specimen cut along the bias direction

**Figure 6.**
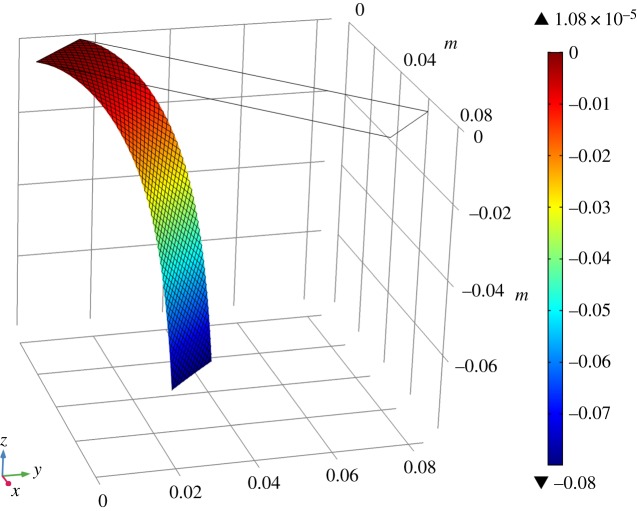
Deformed cantilever strip in bias direction. Colour indicates the out-of-plane displacement. (Online version in colour.)

Finally, we consider a rectangular sample cut along the bias direction of size: *L* = 0.09758 m and *w* = 0.02121 m. Its aspect ratio is λ = 4.60, while the mass density for unit area in the continuum simulation is assumed to be 0.2109 kg m^2^. A new cantilever test has been performed (figure 6), and in order to match the angular deflection of the cantilever end with the predictions of the semi-discrete model (*ϕ* = 62, 6°), we set in the continuum model the torsional stiffness *k*_*T*_ = 1.89 × 10^−4^ Nm.

### (e) Full field out-of-plane measurement and prediction

**Figure 7.**
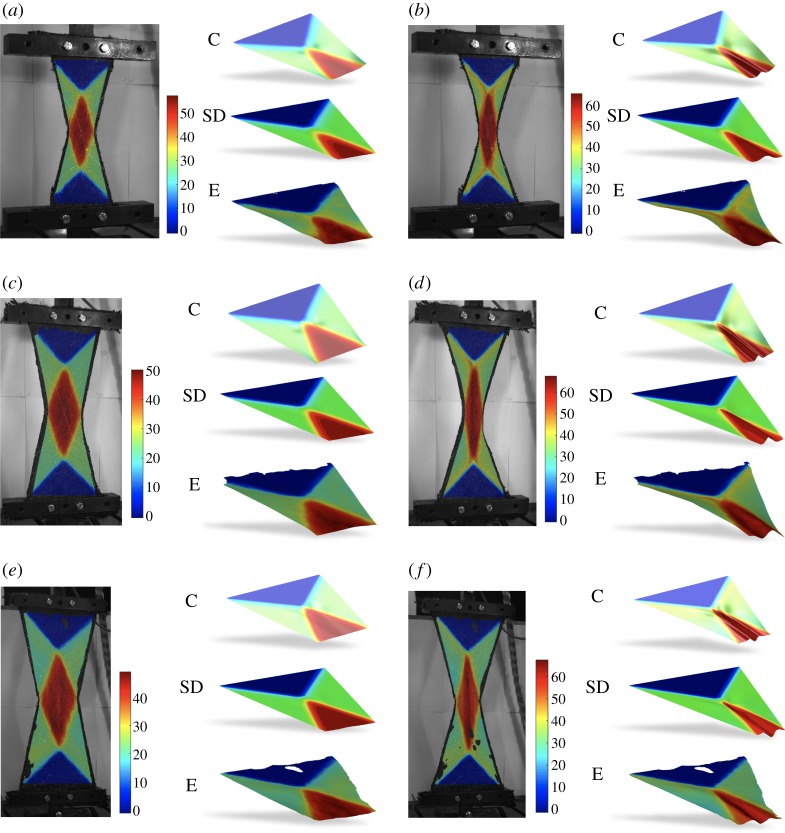
UBE test for the samples of size: (*a*), (*b*) 100 × 200 mm; (*c*), (*d*) 150 × 300 mm; (*e*), (*f* ) 200 × 400 mm (E, experiment; SD, semi-discrete; C, continuum). (*a*) Shear angle approximately 56°, (*b*) 64°, (*c*) 50°, (*d*) 67°, (*e*) 48° and (*f* ) 68°. (Online version in colour.)

The specimens subject to UBE tests in many instances wrinkle when a critical shear angle is reached. To analyse this phenomenon, some numerical simulations are performed using appropriate initial defects to trigger that kind of buckling. In particular, we consider a very small distributed couple on the two long edges in order to force the deformation in one specific direction out-of-plane (such perturbations are required when modelling the test using the implicit continuum approach, in contrast, no such perturbations are required using the explicit semi-discrete approach; here wrinkles are initiated simply from numerical noise). Importantly, the numerical results obtained by both the continuum and semi-discrete models are performed with the material parameters previously identified, thus we can assume these further simulations serve as an independent validation for the two modelling approaches. Figure 7 shows experimental results (from [3]) and numerical results obtained using both the semi-discrete and continuum models for the three specimen sizes. The colour indicates the shear angle (obtained experimentally using digital image correlation combined with the post-processing algorithm discussed in [31]). Each specimen size is shown at two moments in time during the test. One just before and one soon after the onset of wrinkling. Also included are images showing the deformed shape of ‘half samples’ for the continuum model, the semi-discrete model and the experimental test result (top to bottom). The aim here is to clearly show the form of the out-of-plane displacement by viewing the specimen using a mid-section cut, right through the heart of the wrinkle. Clearly, both semi-discrete and continuum models predict similar shapes and wrinkle onset angles as the experimental UBE tests.

**Figure 8.**
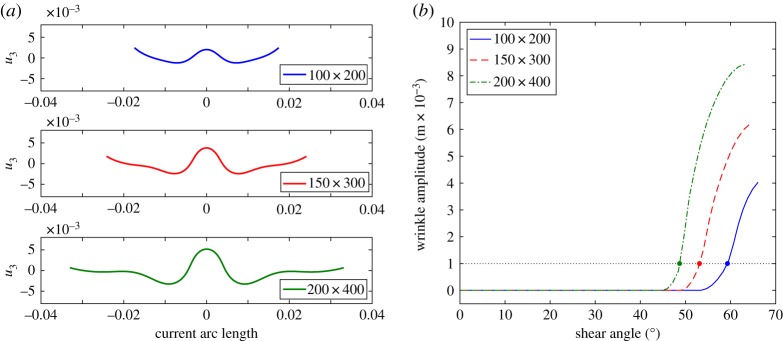
UBE test: wrinkling details by continuum model. (*a*) Out-of-plane displacement along a mid-section for the samples 100 × 200, 150 × 300 and 200 × 400 with shear angles 64°, 67° and 68°, respectively. (*b*) post buckling behaviour. (Online version in colour.)

**Figure 9.**
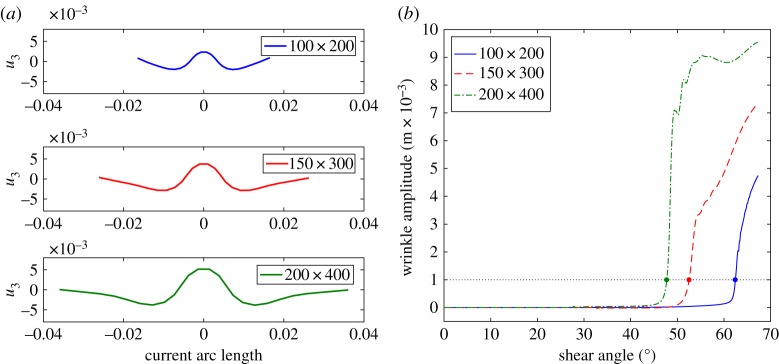
UBE test: wrinkling details by semi-discrete model. (*a*) Out-of-plane displacement along a mid-section for the samples 100 × 200, 150 × 300 and 200 × 400 with shear angles 64°, 67° and 68°, respectively. (*b*) Post buckling behaviour. (Online version in colour.)

To further emphasize this result, figure 8*a* displays the out-of-plane displacement along a mid-section, cut parallel to the short edge of the sample, for different sizes of the samples. Figure 8*b* shows the wrinkle amplitude versus the shear angle at the centre of the sample obtained by the continuum model; figure 9 shows the same plots obtained by the semi-discrete model. These figures allow us to evaluate the wrinkle onset shear angles (given in table 3). The two sets of simulations show similar qualitative behaviour in terms of predicting the size-dependent wrinkle onset angle, though the exact form of the amplitude curves do diverge after wrinkling begins. The reason for the divergence is not entirely clear but is probably related to differences in the stiffnesses at high shear angles. Importantly, both modelling approaches re-produce the inverse relationship between wrinkle onset angle and specimen size observed in the experimental data. It is worth noting that these three critical angles are not directly related to the knee shape in the plots presented in figure 4*a*,*c* and *e*.

**Table 3.** Critical values of shear angle.

sample	100 × 200 mm	150 × 300 mm	200 × 400 mm
angle	61°	53°	47°

**Figure 10.**
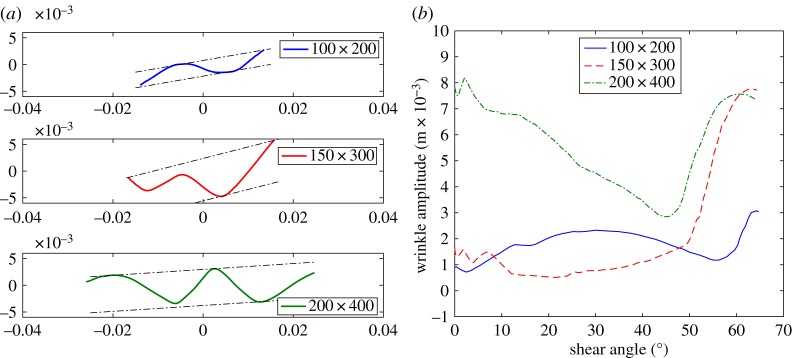
UBE test for the three experimental specimens. (*a*) Profile through mid-section at 63.7°, 68.5° and 67.8° together with two parallel amplitude measurement lines positioned above and below the cross section profile. (*b*) Amplitude versus shear angle, the wrinkle onset angle is identified by the start of the sharp increase in amplitude apparent at higher shear angles. (Online version in colour.)

**Figure 11.**
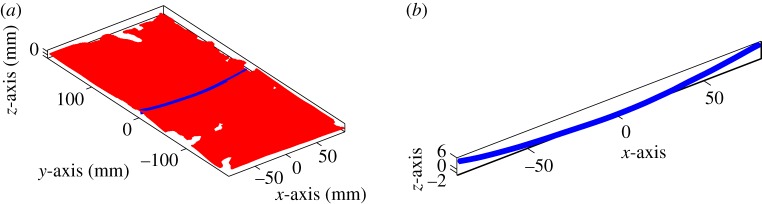
Morphology of 200 × 400 mm specimen at the start of the test. (*a*) Out-of-plane displacement of the whole specimen at the start of the test. (*b*) The profile of the mid-section cross section of the sample showing about 8 mm of out-of-plane displacement. (Online version in colour.)

Analysing wrinkling kinematics is a challenging topic, there is no single robust means of objectively identifying the onset of a wrinkle. In [2,3], the wrinkle onset angle in simulations was defined as the point at which the distance between the peak and trough of the growing wrinkle reached 1 mm. The same definition can be used when analysing the profile of the mid-section of the simulations produced in this investigation (figures 8 and 9). Identifying a similar quantity in experiments is more challenging due to the wide variety of wrinkle morphologies observed in experiments (generated by imperfections in sample preparation, placement and by inherent material variability). One possibility is to analyse cross-sectional information extracted from DIC results using bespoke analysis methods. For example, the ‘wrinkle amplitude’ can be plotted against shear angle by using the distance between two parallel lines positioned above and below the cross-section profile (figure 10*a*). In doing so, the adverse influence of cross-section rotation on the measured wrinkle amplitude is eliminated. Wrinkle amplitude versus shear angle results obtained using this method are shown in figure 10*b*. The results reveal that prior to testing the initial specimen is far from planar and the largest specimen shows the greatest initial out-of-plane displacement. The specimen tends to flatten as the test proceeds (due to tension) then the displacement suddenly increases at the onset of wrinkling. The initial out-of-plane displacement of the sample can appear large when plotted in this way, though figure 11*a*, *b* shows the initial morphology and cross-sectional profile of the mid-section of the 200 × 400 mm specimen are relatively flat despite the large initial out-of-plane displacement. This initial out-of-plane displacement means that a simple displacement-based criteria for wrinkle onset cannot be used when analysing the wrinkle onset angle in experiments. Rather, the experimental wrinkle onset angle can be defined as the shear angle corresponding to the start of the sudden increase in the wrinkle amplitude towards the later stages of the tests, visible in each curve of figure 10*b*. Alternatively, if DIC results are not available, the wrinkle onset angle can simply be defined as the shear angle at which an obvious wrinkle, visible at the end of a test, begins to form and can be identified by visually tracing back the wrinkle to its original perturbation (by reversing the recorded videos). This latter method is inherently subjective as it relies on some interpretation by the observer, though experience suggests that good agreement with the cross-sectional profile method shown in figure 10, is found.

## 5. Conclusion

In this paper, we compare two modelling approaches for engineering fabrics: (i) a semi-discrete model which is characterized by a semi-discrete nature and (ii) a continuum model which is identified by a continuum surface. We denote with the term ‘comprehensive’ for such approaches because we take into account the whole deformation process related to the mechanics of the lattice sub-structure. In particular, during large shear deformations the fibres constituting the fabric are expected to offer resistance to modes of shear, stretching and twisting as well as to any mode of bending, including both of geodesic and of out-of-plane nature. In the semi-discrete model, woven engineering fabrics are assumed to be made by a periodic assembly of unit cells, i.e. pantographic modules which consist of mutually constrained beam and membrane elements connected via torque-free hinge elements. By contrast, the continuum model is a two-dimensional continuum embedded in a three-dimensional space which incorporates the orthotropic symmetry conferred by the initial fibre geometry and is characterized by a deformation energy which depends on both the first- and second-order gradients of displacement. Numerical simulations have been performed using the continuum model with the purpose of identifying the whole set of its material parameters using the predictions of the semi-discrete model as a target for the fitting process. This semi-discrete/continuum identification has been achieved through the design of some *gedanken* experiments that allow us to fit global objective functions representative of the engineering fabric's response. A strong equivalence between the resulting predictions has been shown with both semi-discrete and continuum models successfully predicting the specimen size-dependent shear kinematics and wrinkling behaviour observed in actual experiments. Ultimately, the benefits of the continuum model are aimed at improving computational efficiency and avoiding the need for obstacles such as custom-designed mesh generators and mesh-dependent homogenization (see equations (3.5) and (3.6)). The continuum approach also introduces the possibility of further future gains in computational efficiency through the use of established numerical methods such as adaptive meshing. The main obstacle to the continuum approach is its greater mathematical complexity and consequently the challenges involved in implementing such a model in some commercial finite-element softwares. The approach requires access, not just to the usual state variables such as strain but also the strain gradient. However, this can be done easily with COMSOL Multiphysics using a weak formulation. Irrespective of the relative merits of the two approaches, both the semi-discrete and continuum models have proven themselves to be ‘comprehensive’ and therefore capable of capturing the unusual and potentially important specimen size-dependent mechanical response of engineering fabrics. This ability appears to be fundamental to accurately predicting the wrinkling behaviour of engineering fabrics during large shear deformations.

## Data accessibility

This article has no additional data.
